# Surveillance of HIV and syphilis infections among antenatal clinic attendees in Tanzania-2003/2004

**DOI:** 10.1186/1471-2458-6-91

**Published:** 2006-04-10

**Authors:** Roland O Swai, Geofrey R Somi G, Mecky IN Matee, Japhet Killewo, Eligius F Lyamuya, Gideon Kwesigabo, Tuhuma Tulli, Titus K Kabalimu, Lucy Ng'ang'a, Raphael Isingo, Joel Ndayongeje

**Affiliations:** 1 National AIDS Control Programme, Dar es Salaam, Tanzania; 2 Muhimbili University College of Health Sciences, Dar es Salaam, Tanzania; 3 Commission for Science and Technology, Dar es Salaam, Tanzania; 4 Centres for Disease Control- Tanzania AIDS, Dar es Salaam, Programme, Tanzania; 5 National Institute for Medical Research, Dar es Salaam, Tanzania

## Abstract

**Background:**

This paper presents the prevalence of human immunodeficiency virus (HIV) and syphilis infections among women attending antenatal clinics (ANC) in Tanzania obtained during the 2003/2004 ANC surveillance.

**Methods:**

Ten geographical regions; six of them were involved in a previous survey, while the remaining four were freshly selected on the basis of having the largest population among the remaining 20 regions. For each region, six ANC were selected, two from each of three strata (urban, peri-urban and rural). Three of the sites did not participate, resulting into 57 surveyed clinics. 17,813 women who were attending the chosen clinics for the first time for any pregnancy between October 2003 and January 2004. Patient particulars were obtained by interview and blood specimens were drawn for HIV and syphilis testing. HIV testing was done anonymously and the results were unlinked.

**Results:**

Of the 17,813 women screened for HIV, 1,545 (8.7% (95% CI = 8.3–9.1)) tested positive with the highest prevalence in women aged 25–34 years (11%), being higher among single women (9.7%) than married women (8.6%) (p < 0.07), and increased with level of education from 5.2% among women with no education to 9.3% among those at least primary education (p < 0.001). Prevalence ranged from 4.8% (95% CI = 3.8% – 9.8%) in Kagera to 15.3% (95% CI = 13.9% – 16.8%) in Mbeya and was; 3.7%, 4.7%, 9.1%, 11.2% and 15.3% for rural, semi-urban, road side, urban and 15.3% border clinics, respectively (p < 0.001).

Of the 17,323 women screened for syphilis, 1265 (7.3% (95%CI = 6.9–7.7)) were positive, with highest prevalence in the age group 35–49 yrs (10.4%) (p < 0.001), and being higher among women with no education than those with some education (9.8% versus 6.8%) (p < 0.0001), but marital status had no influence. Prevalence ranged from 2.1% (95% CI = 1.4% – 3.0%) in Kigoma to 14.9% (95% CI = 13.3%-16.6%) in Kagera and was 16.0% (95% CI = 13.3–18.9), 10.5% (95% CI = 9.5–11.5) and 5.8% (95% CI = 5.4–6.3) for roadside, rural and urban clinics, respectively. Syphilis and HIV co-infection was seen in 130/17813 (0.7%).

**Conclusion:**

The high HIV prevalence observed among the ANC clinic attendees in Tanzania call for expansion of current voluntary counselling and testing (VCT) services and access to antiretroviral drugs (ARV) in the clinics. There is also a need for modification of obstetric practices and infant feeding options in HIV infection in order to prevent mother to child transmission of HIV. To increase uptake to HIV testing the opt-out strategy in which all clients are offered HIV testing is recommended in order to meet the needs of as many pregnant women as possible.

## Background

The first AIDS case in Tanzania was reported in the Kagera region in 1983 [1]. In the late 1980's sentinel surveillance activities in antenatal clinics (ANC) were initiated in the Kagera region. Subsequently, in the early 1990s, basing on the assumption that pregnant women attending ANC are representative of the general adult population of both males and females in the reproductive age groups, the NACP developed a protocol for ANC HIV and syphilis sero-surveillance, and expanded activities to 11 of the then 20 regions of mainland Tanzania. This protocol was implemented until 1999, when the NACP undertook a comprehensive review that resulted in improved methods for HIV and syphilis surveillance.

In 1999, the NACP revised the protocol for ANC surveillance and came up with new methods of conducting sentinel surveillance that included the introduction of a 3-month data collection period to replace the previous system of continuous data collection, the introduction of a technology of using dried blood spots (DBS) filter paper cards for blood storage and the standardization of HIV test approaches and quality assurance system.

The first ANC surveillance that used this strategy was conducted in 2000/2001.

The second survey using the new approach was conducted between October 2003 and January 2004 at 57 ANC sites located in ten regions of Tanzania. Similar to the previous sero-surveillance round [2], clinics in selected regions were stratified into three strata of urban, peri-urban and rural to allow the output to be presented according to these strata.

The paper presents the prevalence of human immunodeficiency virus (HIV) and syphilis infections among women attending antenatal clinics (ANC) in Tanzania obtained during the 2003/2004 ANC surveillance.

## Methods

A sero-survey was conducted between October 27, 2003 and January 23, 2004 among pregnant women attending selected ANC clinics in Tanzania. This was the second of a series of sentinel sero-surveys involving pregnant women that was started in 2001/2002. The methods used in the two surveys were similar.

### Site selection and study population

In 1996 Tanzania Demographic and Health Surveys (DHS) clustered regions of Tanzania mainland into six geographical zones [3]. The six zones were Coastal, Northern Highland, Lake, Central, Southern Highland and Southern. Site selection for the 2001/2 surveillance round was based on the location of the six zones from each of which one region was selected on the basis of prior participation in national ANC sentinel surveillance studies and availability of relevant data on HIV prevalence. For the current survey-round (2003/4), ten regions were selected, six of them being those surveyed in 2001/2002 while the remaining four were freshly selected on the basis of having the largest population among the remaining regions. Thus, regions with largest populations were preferentially selected.

Subsequent to the selection of the ten regions, six ANC clinic sites were selected from each region on the basis of a large catchment population, high average number of monthly bookings, road accessibility and previous participation in national sentinel surveillance studies. Hence, a total of sixty sites were obtained. However, three of the sites did not participate in the training exercise for the surveillance protocol and were therefore excluded from the study. The 57 sites included in the study were stratified into three distinct geographical categories for each region according to whether they were urban, peri-urban or rural with the following definition:

• Urban – ANC clinic is located within the regional headquarter town

• Peri-urban – ANC clinic is located in a town other than the regional headquarter town

• Rural – ANC clinic is located in a rural area.

Peri-urban sites located along major roads that cross either a border town or the region were classified as border sites or roadside sites, respectively. The study population consisted of all the pregnant women presenting for the first time to the selected ANC clinics for any pregnancy during the surveillance period of three months.

### Specimen collection and transportation

After obtaining an informed verbal consent from the pregnant woman at the clinic, about 3–5 ml of blood was collected aseptically by venepuncture in EDTA vacutainer tubes for RPR testing. The syphilis result was recorded on the woman's clinic card and on the surveillance data collection form. The remaining blood specimen was then used to prepare DBS for HIV testing whereby consent was not needed. ANC staff applied 100 μl of whole blood to each of five circles on a DBS card (labeled with a unique surveillance number), which was then left to dry at room temperature. At this point the upper part of the surveillance data form that contains a woman's clinic card number is removed and destroyed. ANC staff mailed completed data collection forms and DBS for HIV testing using weekly courier service to the National HIV Reference Laboratory (NHRL) at MUCHS.

### Syphilis testing and treatment

In all study sites rapid plasma reagin (RPR) test was done on the site. In the majority of rural sites, ANC nurse performed the test whereas, in most of the urban and peri-urban sites it was carried out in a laboratory by a Laboratory Technician/Assistant. Results were recorded directly on the data collection form and on woman's clinic card or laboratory investigation request form. No TPP or TPHA was done on the positives RPR samples for confirmation of test results or quality assurance. Women whose RPR test results were positive were offered treatment based on the National STD Treatment Guidelines [4].

### HIV testing

At the National HIV Reference laboratory, dried blood was eluted from the DBS card and tested using Vironostika^® ^HIV Uni-Form II Ag/Ab ELISA test (Biomerieux, The Netherlands). Specimens with negative results underwent no further testing and were considered negative. Specimens positive on the first ELISA were retested using a second ELISA test, Vironostika^® ^HIV Uni-Form II Plus O (Biomerieux, The Netherlands) on the same DBS card. The ELISA algorithm was independently validated by CDC in Atlanta and by the NHRL at MUCHS. Specimens that reacted positive on the second test were considered positive. Specimens that reacted negative on the second test were considered negative.

### Quality assurance

Ten per cent of all samples were randomly selected by MUCHS for quality assurance (QA) testing at a laboratory different from the one which carried out the initial testing. Every 10th sample starting from number 01 at each site was selected.

### Ethical considerations

The Ministry of Health awarded ethical clearance to the National HIV Surveillance protocol. Because HIV test results were not linked by name and tests were performed on residual blood from routine syphilis screening, obtaining informed consent was not warranted. All information linking the sample to the client was removed and DBS HIV testing occurred anonymously. Women who wished to know their HIV status were referred to a nearby voluntary counseling and testing (VCT) facility.

### Data entry and analysis

Laboratory technologists entered HIV test results in laboratory log books and delivered them to NACP. NACP data entry clerks performed double entry of data manually into Epi-Info Programme [5]. The two files were validated and incorrect entries were corrected.

Data were analysed by a team of researchers from NACP, MUCHS, NIMR and CDC, initially during a weeklong analysis meeting, followed by several rounds of consensus gathering and review. ANC HIV and syphilis prevalence rates were calculated by age, marital status, parity, educational level, and distance from residence to ANC and duration in residence. Prevalence were calculated with 95% confidence intervals (CI) to guide interpretation. Data were analysed using the statistical software packages EpiInfo 2002 [5] and Stata for windows Version 8.0 [6].

## Results

### HIV prevalence

**Figure 1:**
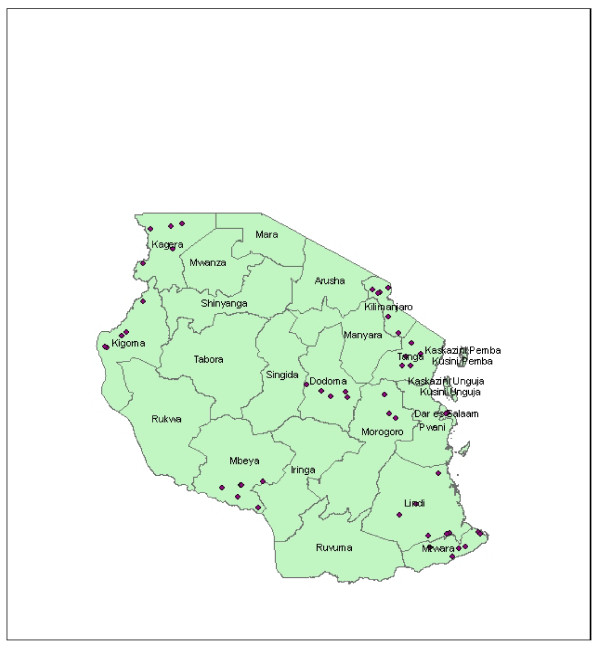
Map of Tanzania study showing study sites.

A total of 17,813 antenatal clinic attendees were enrolled in the ANC serosurveillance study from 57 clinics located in 10 regions of Tanzania between October 27^th ^2003 and January 23^rd ^2004. The number of enrolled women regionally ranged from 1135 in Lindi to 3018 in Dar es Salaam. A total of 1,545 women tested HIV positive resulting in an overall HIV prevalence in this population of 8.7% (95% CI = 8.3, 9.1). HIV infection prevalence ranged from a low of 4.7% (95% CI = 3.8, 9.8%) in Kagera region to a high of 15.7% (95% CI = 13.9–16.8) in Mbeya region (Fig 1). HIV prevalence is also presented at the clinic level (Table 1).

Of the 57 ANC sites surveyed in this study, 13 (22.8%) were found to have a prevalence of HIV infection of 10% or more. These high prevalence sites consisted of one clinic in Dodoma (urban), three urban clinics in Dar es Salaam, two clinics in Lindi (urban), one in Morogoro (urban), one in Tanga (urban) and five in Mbeya (2 urban, 1 rural, 1 border and 1 road side) (Table 1).

**Figure 2:**
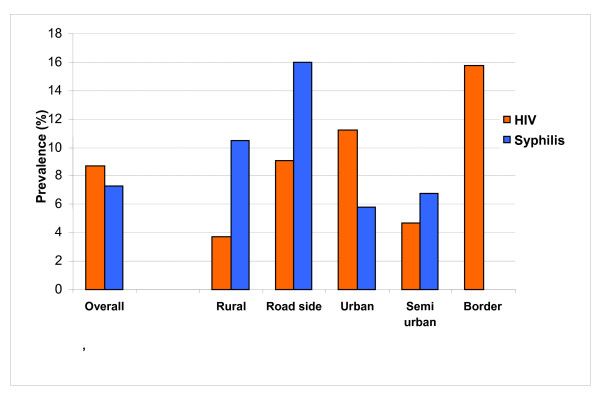
Prevalence of HIV and syphilis among ANC attendees by ANC location.

The HIV prevalence differed according to residence ranging between 3.7% for rural clinics, 4.7% for semi-urban, 9.1% for road side, 11.2% for urban and 15.3% for border clinics (p < 0.001) (Figure 2).

**Figure 3:**
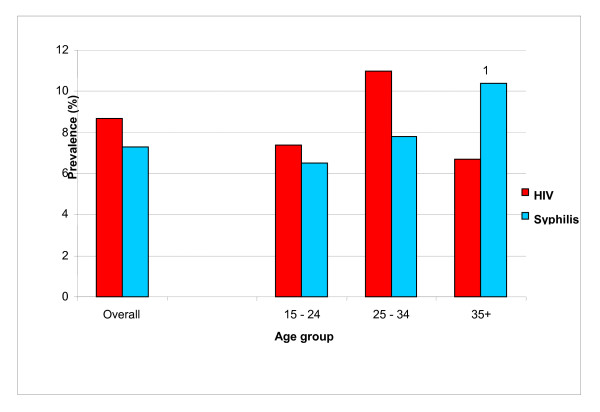
Prevalence of HIV and syphilis by age group among ANC attendees, Tanzania

**Figure 4:**
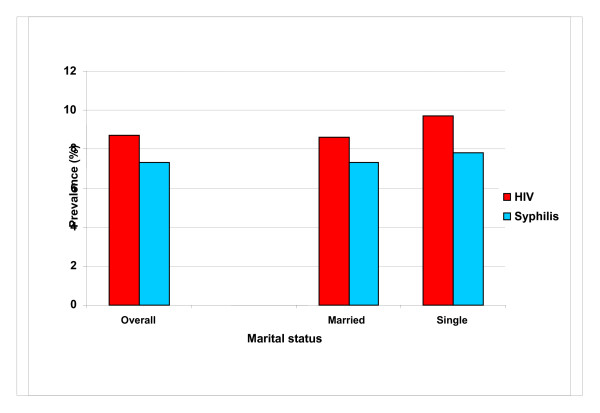
Prevalence of HIV and syphilis among ANC attendees by marital status.

In all regions, HIV prevalence was highest among women aged 25 – 34 years. Rates were similar among the youngest and oldest age groups (Fig 3). HIV prevalence among single women (9.7%) was slightly higher but not statistically different from that of married women (8.6%) (p < 0.07), (Fig 4).

**Figure 5:**
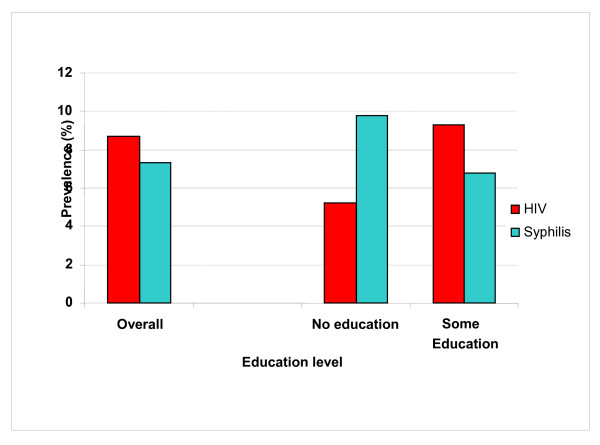
Prevalence of HIV and syphilis among ANC Attendees by education status, Tanzania, 2003/04.

HIV prevalence increased with level of education from 5.2% among women with no education to 9.3% among those with some primary education or more (p < 0.001) (Fig 5).

### Syphilis prevalence

A total of 17,323 ANC attendees were tested for syphilis during the study period. A total of 1,265 women tested positive, an overall syphilis prevalence of 7.3% (95% CI = 6.9–7.7) (Fig 1). Syphilis infection prevalence ranged from a low of 2.1% (95% CI = 1.4,3.0) in Kigoma region to a high of 14.9% (95% CI = 13.3,16.6) in Kagera region where interestingly, the HIV prevalence was found to be the lowest (Fig 1).

**Figure 6:**
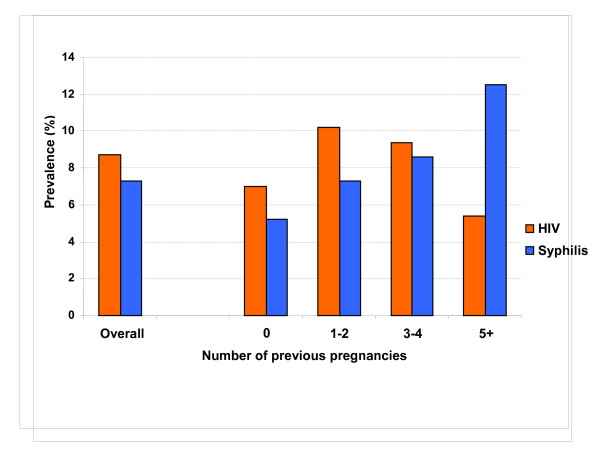
Prevalence of HIV and syphilis among ANC attendees by number of previous pregnancies, Tanzania, 2003/04.

The prevalence of syphilis was highest among attendees from roadside clinics 16.0% (95% CI = 13.3–18.9) than those from rural clinics 10.5% (95% CI = 9.5–11.5) and lowest among urban clinic attendees 5.8% (95% CI = 5.4–6.3) (Fig 2). Women living in rural areas had higher prevalence than those in urban areas (p < 0.0001) (Figure 6). Marital status did not appear to influence the prevalence of syphilis (Fig 4). The age specific prevalence of syphilis were 6.5% for age group 15–24, 7.8% for age group 25–34 and 10.4% for age group 35–49 (Fig 3). The observed differences in age-specific prevalence were statistically significant (p < 0.001), suggesting that there was a higher likelihood of having syphilis for women aged >34 years compared to those less than 34 years of age (Fig 3). As in previous years, in contrast to women with HIV, women with no education were more likely to be infected with syphilis than were women with some education (p < 0.0001) (Fig 5). Overall, 130/17,813 (0.7%) of clinic attendees were co-infected with syphilis and HIV.

## Discussion

The prevalence of HIV infection in this surveillance round was 8.7%, which compares with the findings of a population survey showing the prevalence of HIV to be 7.3% among adult Tanzanians aged 15–49 years [7]. The peak age was 25 to 34 years, which may explain the highest HIV prevalence among women with one or two previous pregnancies compared to those with more (Fig 6).

HIV infection show strong regional variations, with Mbeya being the most affected (15.7%) and Dar es Salaam (10.8%), the capital city, being second most affected region. These data are in keeping with the previous ANC surveillance data (Table 2) and recent reports of the general population survey, showing that these regions have the highest HIV prevalence in Tanzania[7]. Remarkably, Kagera, a region where the HIV/AIDS epidemic started in Tanzania and where the prevalence of HIV was high for sometime[8] had the lowest prevalence of HIV infection, an observation also been seen in population surveys [7].

Significantly, the HIV prevalence of women attending road side, border and urban ANC clinics was higher than rural attendees, an observation that is consistent with past [8-11] and recent studies [7]. These differences have been linked with differences in community factors such as the level of trade, social and economic activity [12], ratio of bar workers per male population, level of community mobility, and distance to the nearest town [16]. There was slightly higher prevalence of HIV among single than married women, which was statistically not significant, which is in keeping with the previous ANC surveillance report (Table 2). Other studies in Tanzania have found highest HIV prevalence among married women or formerly married women [7,9]. Such variations may imply that marital status per se is not an indicator of sexual activity and hence risk for HIV infection, which indicate the need to consider risk of their partners as well [14].

This surveillance showed that HIV prevalence increased with the level of education, from 5.2% among women with no education to 9.3% with women with primary or higher level of education, which was also observed in the previous ANC surveillance (Table 2) and other studies [7,14]. This could be due to the fact the educated, through their access to income and their position in society, engage in sexual behaviours which place themselves and their spouses at risk of HIV infection. The high level of HIV prevalence among the educated individuals will lead to substantial economic losses through the erosion of Africa's most able and most educated segment of the population.

When the HIV results of this surveillance are compared with those of the first ANC survey conducted in 2001/2002 (Table 2) we notice a decrease in prevalence in Dar es Salaam and Mtwara, with the other regions have no change. The HIV prevalence among women attending border clinics fell from 17.3% to 15.3%. These changes were rather small and are probably a result of random variation in the different regions and sites. As with the previous ANC surveillance, the age of peak HIV prevalence was 25–34 years compared with 30–34 for females in the population survey.

The overall prevalence of syphilis was 7.3%, which compares with 8.2% reported in the last surveillance conducted in 2001/2002 (Table 2). Like HIV, the syphilis data show strong geographical variations, ranging from 2.1% in Kigoma to 14.9% in Kagera. There were also notable observations from the syphilis data including higher syphilis prevalence in women from roadside clinics. Unlike HIV, syphilis was higher among women without formal education and those aged ≥ 34 years, and there was no association with marital status. These observations are in keeping with previous ANC surveillance reports (Table 3). Collectively these findings indicate that syphilis is associated with poverty, inadequate access to health care, and lack of education.

Remarkably, some regions with low HIV prevalence e.g. Kagera had very high prevalence of syphilis, and vice-versa for Mbeya, Dar es Salaam and Tanga regions and there were also very significant variations even in areas that were geographically close to each other, such as the studied areas in Dar es Salaam, Dodoma and Kagera, without corresponding differences in HIV prevalence (Table 1). The syphilis data need to be interpreted with caution for several reasons including; 1) the use of RPR without confirmatory testing with a more specific test for syphilis such as FTA-ABS or TPHA, testing [15] or antitreponemal IgM ELISAs [16] 2) lack of quality control measures 3) although RPR test is easy to perform and inexpensive it may be difficult to interpret and requires training of health personnel (West et al), which may have been especially the case in the rural areas where testing was done by inexperienced ANC nurses, 4) false negatives may occur both in early primary cases and in patients with secondary syphilis as a result of prozone reactions (Berkowitz 1990), and 5) poor performance of RPR test under field conditions that are typical of many developing country health centres [17-19] due to temperature, dust and the light conditions [17,20,21]. Future surveillance for syphilis infection should address these issues in order to improve the quality of the syphilis data.

## Conclusion

The high HIV prevalence observed among the ANC clinic attendees in Tanzania call for increase in voluntary counselling and testing (VCT) services and access to antiretroviral drugs (ARV) in the clinics. There is also a need for modification of obstetric practices and infant feeding options in HIV infection in order to prevent mother to child transmission of HIV. To increase uptake to HIV testing the opt-out strategy in which all clients are offered HIV testing is recommended. Post-test counselling of HIV-negative women should also be carried out to prevent new HIV infections. To this end we are suggesting; i) scaling up existing prevention of mother-to-child transmission (PMTCT) programs by rapidly mobilizing resources, ii) providing technical assistance and expanded training for health care providers (including family planning providers, traditional birth attendants, and others) on appropriate antenatal care, safe labor and delivery practices and breast-feeding, iii) strengthening the referral links among health care facilities and providers, iv) ensuring effective supply chain management of the range of PMTCT-related products and equipment, and v) expanding PMTCT programs to include ARV treatment for eligible HIV-infected mothers and other members of the child's immediate family (commonly known as "PMTCT-plus").

## Competing interests

The author(s) declare that they have no competing interests.

## Authors' contributions

Authors contributed equally to this work. All authors read and approved the final manuscript.

## Pre-publication history

The pre-publication history for this paper can be accessed here:



## Figures and Tables

**Table 1:** Prevalence of HIV and syphilis infection by ANC sites, Tanzania 2003/04

	***RPR***				***HIV***			
***Site***	***Total***	***Positive***	***% Prevalence***	***95% CI***	***Total***	***Positive***	***% prevalence***	***95% CI***
***Dar es salaam***	***2959***	***151***	***5.1***	**4.3 – 6.0**	***3018***	***325***	***10.8***	**9.7 – 11.9**
^c^Buguruni	879	11	1.3	0.6 – 2.2	884	107	12.1	10.0 – 14.4
^c^Kasorobo	446	46	10.3	7.7 – 13.5	450	43	9.6	7.0 – 12.7
^c^Kigamboni	386	4	1.0	0.3 – 2.6	389	36	9.3	6.6 – 12.6
^c^Oysterbay	466	37	7.9	5.7 – 10.8	478	50	10.5	7.9 – 13.6
^c^Kimara	361	10	2.8	1.3 – 5.0	384	35	9.1	6.4 – 12.4
^c^Kiwalani	421	43	10.2	7.5 – 13.5	433	54	12.5	9.5 – 16.0
								
***Dodoma***	***1597***	***134***	***8.4***	**7.1 – 9.9**	***1621***	***126***	***7.8***	**6.5 – 9.2**
^a^Bahi	176	6	3.4	1.3 – 7.3	181	8	4.4	1.9 – 8.5
^a^Handali	228	64	28.1	22.3 – 34.4	228	1	0.4	0.0 – 2.4
^e^Kibaigwa	203	5	2.5	0.8 – 5.7	203	18	8.9	5.3 – 13.7
^c^Makole	331	4	1.2	0.3 – 3.1	339	31	9.1	6.3 – 12.7
^b^Mpwapwa	308	29	9.4	6.4 – 13.2	319	19	5.9	3.6 – 9.1
^c^Wajenzi	351	26	7.4	4.9 – 10.7	351	49	13.9	10.5 – 18.0
								
***Kagera***	***1814***	***270***	***14.9***	**13.3 – 16.6**	***1826***	***87***	***4.7***	**3.8 – 5.8**
^c^Bukoba	409	21	5.1	3.2 – 7.7	410	39	9.5	6.9 – 12.8
^b^Katoro	244	64	26.2	20.8 – 32.2	245	6	2.4	0.9 – 5.3
^a^Kimeya	235	51	21.7	16.6 – 27.5	241	2	0.8	0.1 – 3.0
^a^Nkwenda	451	86	19.1	15.5 – 23.0	452	7	1.5	0.6 – 3.2
^b^Nyamiaga	180	27	15.0	10.1 – 21.1	182	4	2.2	0.6 – 5.5
^c^Rwamishenye	295	21	7.1	4.5 – 10.7	296	29	9.8	6.7 – 13.8
								
***Kilimanjaro***	***1405***	***89***	***6.3***	**5.1 – 7.7**	***1429***	***81***	***5.7***	**4.5 – 7.0**
^e^Hedaru	219	84	38.4	31.9 – 45.1	221	5	2.3	0.7 – 5.2
^c^Majengo	323	1	0.3	0.0 – 1.7	323	22	6.8	4.3 – 10.1
^a^Masama	266	0	0	0.0 – 1.4	268	17	6.3	3.7 – 10.0
^a^Umbwe	103	0	0	0.0 – 3.5	103	3	2.9	0.6 – 8.3
^b^Huruma	187	2	1.1	0.1 – 3.8	323	26	8.1	5.3 – 11.6
^c^Pasua	307	2	0.7	0.1 – 2.3	191	8	4.2	1.8 – 8.1
								
***Mbeya***	***2386***	***149***	***6.2***	**5.3 – 7.3**	***2442***	***384***	***15.7***	**14.3 – 17.2**
^e^Chimala	249	18	7.2	4.3 – 11.2	249	38	15.3	11.0 – 20.3
^a^Ilembo	178	13	7.3	3.9 – 12.2	188	15	8.0	4.5 – 12.8
^c^Kiwanjampaka	724	29	4.0	2.7 – 5.7	726	137	18.9	16.1 – 21.9
^d^Kyela	315	22	7.0	4.4 – 10.4	323	51	15.8	12.0 – 20.2
^a^Igamba	195	29	14.9	10.2 – 20.7	228	24	10.5	6.9 – 15.3
^c^Ruanda	725	38	5.2	3.7 – 7.1	728	116	15.9	13.3 – 18.8
								
***Mtwara***	***1262***	***115***	***9.1***	**7.6 – 10.8**	***1279***	***66***	***5.1***	**4.0 – 6.5**
^c^Ligula	388	29	7.5	5.1 – 10.6	389	37	9.5	6.8 – 12.9
^b^Mangaka	264	21	8.0	5.0 – 11.9	267	13	4.9	2.6 – 8.2
^a^Nanyamba	173	9	5.2	2.4 – 9.6	176	6	3.4	1.3 – 7.3
^b^Tandahimba	146	8	5.5	2.4 – 10.5	147	1	0.7	0.0 – 3.7
^c^Likombe	165	29	17.6	12.1 – 24.3	165	7	4.2	1.7 – 8.5
^a^Mkunya	126	19	15.1	9.3 – 22.5	135	2	1.5	0.2 – 5.2
								
***Kigoma***	***1246***	***26***	***2.1***	**1.4 – 3.0**	***1349***	***69***	***5.1***	**4.0 – 6.4**
^b^Kibondo	204	0	0.0	0.0 – 1.8	217	7	3.2	1.3 – 6.5
^b^Kiganamo	293	5	1.7	0.6 – 3.9	326	11	3.4	1.7 – 6.0
^c^Kigoma	296	6	2.0	0.7 – 4.4	301	21	7.0	4.4 – 10.5
^a^Nyakitonto	113	2	1.8	0.2 – 6.2	163	6	3.7	1.4 – 7.8
^c^Ujiji	340	13	2.8	2.1 – 6.4	342	24	7.0	4.5 – 10.3
								
***Lindi***	***1112***	***79***	***7.1***	**5.7 – 8.8**	***1135***	***80***	***7.1***	**5.6 – 8.7**
^a^Chumo	132	4	3.0	0.8 – 7.6	141	4	2.8	0.8 – 7.1
^b^Liwale	296	7	2.4	1.0 – 4.8	307	8	2.7	1.1 – 5.1
^b^Nachingwea	227	32	14.1	9.8 – 19.3	227	22	9.7	6.2 – 14.3
^a^Nyangao	231	20	8.7	5.4 – 13.1	233	12	5.2	2.7 – 8.8
^c^Sokoine	117	7	6.0	2.4 – 11.9	119	16	13.5	7.9 – 20.9
^c^Town clinic	109	9	8.3	3.8 – 15.1	113	18	15.9	9.7 – 24.0
								
***Morogoro***	***1556***	***187***	***12.0***	**10.4 – 13.7**	***1630***	***147***	***9.0***	**7.7 – 10.5**
^a^Hembeti	81	9	11.1	5.2 – 20.0	99	2	2.1	0.2 – 7.1
^a^Mkuyuni	154	5	3.3	1.1 – 7.4	173	3	1.7	0.4 – 5.0
^c^Morogoro	550	54	9.8	7.5 – 12.6	559	64	11.5	8.9 – 14.4
^c^Uhuru	771	119	15.4	13.0 – 18.2	799	78	9.8	7.8 – 12.0
								
***Tanga***	***1986***	***65***	***3.3***	**2.5 – 4.2**	***2084***	***191***	***9.2***	**8.0 – 10.5**
^b^Handeni	380	8	2.1	0.9 – 4.1	403	32	7.9	5.5 – 11.0
^a^Kwamkono	158	16	10.1	5.9 – 15.9	168	8	4.7	2.1 – 9.2
^b^Lushoto	237	1	0.4	0.0 – 2.3	246	13	5.3	2.8 – 8.9
^a^Magoma	171	7	4.1	1.7 – 8.3	175	2	1.1	0.1 – 4.1
^c^Makorola	518	8	1.5	0.7 – 3.0	545	36	6.6	4.7 – 9.0
^c^Ngamiani	522	25	4.8	3.1 – 69.9	547	100	18.3	15.1 – 21.8

Key: ^a^rural, ^b^semi-urban, ^c^urban, ^d^border, ^e^roadside

**Table 2:** HIV data of the ANC surveillance studies conducted in 2001/2002 and 2003/2004

	**ANC Surveillance 2003/2004**	**ANC Surveillance 2001/2002**
**Region**		
Dar es Salaam	10.8%	12.8%
Dodoma	7.8%	6.1%
Kagera	4.7%	5.6%
Kilimanjaro	5.7%	6.3%
Mbeya	15.7%	16%
Mtwara	5.1%	7.1%
Kigoma	5.1%	ND
Lindi	7.1%	ND
Morogoro	9.0%	ND
Tanga	9.2%	ND
		
**Residence**		
Urban	11.2%	12.1%
Semi-urban	4.7%	3.7%
Roadside	9.1%	10.3%
Border	15.3%	17.3%
Rural	3.7%	4.1%
**Peak age**	25–34	25–34
		
**Education**		
None	5.2%	6.4%
Some education	9.3%	10.2%
		
**Marital status**		
Married	8.6%	9.1%
Single	9.7%	12.6%
**Overall Prevalence**	8.7%	9.6%

ND = No Data

**Table 3:** Syphilis data of the ANC surveillance studies conducted in 2001/2002 and 2003/2004

	**ANC Surveillance 2003/2004**	**ANC Surveillance 2001/2002**
**Region**		
Dar es Salaam	5.1%	4.8%
Dodoma	8.4%	6.1%
Kagera	14.9%	9.3%
Kilimanjaro	6.3%	3.0%
Mbeya	6.2%	10.7%
Mtwara	9.1%	10.5%
Kigoma	2.1%	ND
Lindi	7.1%	ND
Morogoro	12.0%	ND
Tanga	3.3%	ND
		
**Residence**		
Urban	5.8%	5.1%
Semi-urban	6.8%	13.3%
Roadside	16.0%	9.7%
Border	7.0%	13.4%
Rural	10.5%	4.1%
**Peak age**	+35	+ 35
		
**Education**		
None	9.8%	11.5%
		
Some education	6.8%	7.5%
		
**Marital status**		
Married	7.3%	8.2%
Single	7.8%	8.2%
**Overall Prevalence**	7.3%	7.2%

ND = No Data
